# Functional adaptation of the infant craniofacial system to mechanical loadings arising from masticatory forces

**DOI:** 10.1098/rspb.2024.0654

**Published:** 2024-06-19

**Authors:** Ce Liang, Federica Landi, Izel Ezgi Çetin, Antonio Profico, Costantino Buzi, Hugo Dutel, Roman Hossein Khonsari, Paul O'Higgins, Mehran Moazen

**Affiliations:** ^1^ Department of Mechanical Engineering, University College London, London WC1E 7JE, UK; ^2^ Institut Català de Paleoecologia Humana i Evolució Social (IPHES-CERCA), Tarragona 43007, Spain; ^3^ Departament d’Història i Història de l’Art, Universitat Rovira i Virgili, Tarragona 43002, Spain; ^4^ Department of Oral and Maxillofacial Surgery, Erasmus Medical Centre, Rotterdam 3015, The Netherlands; ^5^ Craniofacial Growth and Form Laboratory, Hôpital Necker–Enfants Malades, Assistance Publique - Hôpitaux de Paris, Faculté de Médecine, Université Paris Cité, Paris 75015, France; ^6^ Department of Biology, University of Pisa, Pisa 56126, Italy; ^7^ Bristol Palaeobiology Group, School of Earth Sciences, University of Bristol, Bristol S8 1TQ, UK; ^8^ Université de Bordeaux, CNRS, MCC, PACEA, UMR 5199, Pessac 33600, France; ^9^ Department of Archaeology and Hull York Medical School, University of York, York YO10 5DD, UK

**Keywords:** masticatory muscle, temporal fascia, bite force, anatomical cross-sectional areas, finite-element analysis, computational biomechanics

## Abstract

The morphology and biomechanics of infant crania undergo significant changes between the pre- and post-weaning phases due to increasing loading of the masticatory system. The aims of this study were to characterize the changes in muscle forces, bite forces and the pattern of mechanical strain and stress arising from the aforementioned forces across crania in the first 48 months of life using imaging and finite element methods. A total of 51 head computed tomography scans of normal individuals were collected and analysed from a larger database of 217 individuals. The estimated mean muscle forces of temporalis, masseter and medial pterygoid increase from 30.9 to 87.0 N, 25.6 to 69.6 N and 23.1 to 58.9 N, respectively (0–48 months). Maximum bite force increases from 90.5 to 184.2 N (3–48 months). There is a change in the pattern of strain and stress from the calvaria to the face during postnatal development. Overall, this study highlights the changes in the mechanics of the craniofacial system during normal development. It further raises questions as to how and what level of changes in the mechanical forces during the development can alter the morphology of the craniofacial system.

## 1. Introduction

The human craniofacial system consists of several bony elements, connected via fibrous and cartilaginous joints, housing sensory organs such as the brain and eyes and supporting the critical functions of the masticatory and respiratory systems [1–3]. The functioning of the masticatory apparatus relies on four major components: the bones (cranium and mandible), muscles (temporalis, masseter and lateral and medial pterygoid), teeth and associated soft tissues such as the tongue and lips [4]. Mastication is a rhythmical function executed through muscles connecting the cranium and mandible, where the temporalis, masseter and medial pterygoid are major jaw-closing muscles, while the lateral pterygoid is a major jaw-opening muscle [5]. The masticatory apparatus forms a rather robust musculoskeletal structure, enabling us to chew and bite a wide range of foods [4,6,7].

It is evident that the mechanical stimuli arising from masticatory actions result in functional adaptation of the developing cranium, at least to some degree [8–13]. From 0 to 48 months, the infant cranium undergoes rapid morphological changes in size and shape [14,15]. It is well accepted that the infant cranium alters in response to, and to accommodate, the volumetric changes of key organs and cavities [3,14,16]. This involves displacement, expansion and modelling of skeletal elements [7,17], as well as tissue differentiation across the craniofacial sutures [2,18–21]. Recently, Haravu *et al*. [22] reported the potential impact of masticatory system loading on the closure of the metopic suture. Nonetheless, our fundamental understanding of the biomechanical changes in the human masticatory system during postnatal development, especially in the first few years after birth, is still limited. For example, at the system level, we are still questioning what drives the growth of the face. If this is mainly driven by masticatory system muscle and reaction forces, then how do mechanical strains and stresses that the craniofacial system, and more specifically the face, experience change with the increasing muscle forces and bite forces during the early stages of postnatal growth and development (e.g. 0–48 months of age).

Several approaches have been used to characterize changes in the masticatory system. Bite force has been measured using a pressurized deformable tube connected to force sensors. Using such a system, *in vivo* experimental bite force data have been reported after 36 months of age (post-weaning) [4,23–27], when the deciduous dentition has erupted and in occlusion [28,29] enabling biting on solid food [30]. Masticatory system muscle peak forces are generally estimated based on their cross-sectional areas (CSAs) measured from clinical computed tomography (CT) images. The resolution of these images limits which muscles can be identified and virtually sectioned in appropriate orientations [3,8,9,22,31]. Therefore, data on masticatory muscle forces are scarce in infants.

Computational methods such as finite-element analyses (FEA) have been widely used to investigate the functional biomechanics of the human masticatory system [8,12,13,22,32–35] and can provide insights into masticatory muscle forces, maximum bite forces and joint reaction forces as well as strain–stress regimens across the skeleton. These data are difficult, if not impossible, to obtain from *in vivo* experiments in humans [36] but can be approached via FEA. However, masticatory system musculature is inevitably highly simplified in FEA studies, being modelled, for example, by applying single or multiple force vectors from muscle origins to insertions [22,32] and omitting the effects of muscle fibre arrangement. Thus, FEA might be expected to underestimate muscle and bite forces, but in several previous studies, the errors are within an acceptable range [13,37,38]. Furthermore, it is common to omit the temporal fascia in FEA models of the human masticatory system. However, inclusion of the temporal fascia in a model of macaque [10] has been shown to minimize the often observed large inferior deflection of the zygomatic arche and the magnitude of strains across the arch [12,32,33,35], i.e. stabilizing the zygomatic arch against the forceful downward loads arising from the masseter muscle.

This article presents the first detailed and longitudinal FEA of the impact of the actions of the masticatory muscles and temporal fascia on the infant cranium throughout the first 4 years of life. It investigates the effects of differences in the masticatory system loading during the pre- and post-weaning phases. The aims of this study are to (i) estimate masticatory system muscle forces using anatomical CSAs; (ii) predict temporal fascial force and bite forces during three different bite modes; and (iii) predict the distributions and magnitudes of mechanical strains and stresses over the crania arising from masticatory system loading. It is anticipated that these will enable us to better understand the mechanical influences on craniofacial growth. This will contribute to better modelling of craniofacial growth, which can be applied to advance treatment of conditions affecting this system (e.g. craniosynostosis).

## 2. Material and methods

### 2.1. Data collection

**Figure 1.**
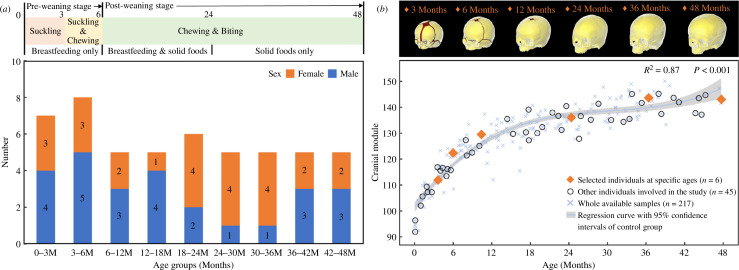
Details of the head CT dataset of normal individuals and average skull models used in this study. (*a*) Sample distribution of a total of 51 normal individuals according to specific age groups from 0 to 48 months. Masticatory behaviours at pre- and post-weaning stages throughout the studied ages are presented at the top. (*b*) Highlights of the selected six individuals that were used to develop finite element models in this study, i.e. at 3, 6, 12, 24, 36 and 48 months of age. The *in silico* skull models are shown at the top with segmented bones (in yellow) and sutures (in red). See electronic supplementary material, figure S1 for more details of the *in silico* models. Cranial module versus age in months is plotted in the lower frame for the whole dataset.

From an available dataset (*n* = 217) [14,39], a total of 51 CT scans of the heads of normal individuals (age: 0–48 months) were selected, by identifying one or two individuals to represent each month. The main selection criteria for the CT scans of these individual cases were minimal noise and image distortion and that they were of adequate quality to locate the soft tissues and their boundaries (figure 1*a*; see electronic supplementary material, table S1 for the voxel sizes of selected CT scans). All CT data were anonymized and provided by the Necker–Enfants Malades University Hospital in Paris, France (study no. 2018RK18). These individuals were born between 2008 and 2018 and scanned for clinical purposes to investigate minor trauma, head and neck acute infections or febrile seizures.

### 2.2. Image processing and model development

*In vivo* skull models were reconstructed from CT scans of the selected individuals (*n* = 51) using Avizo (V2022.1, Thermo Fisher Scientific, Waltham, MA, USA). Six skulls were selected from the above reconstructions as closely representing the mean sizes and shapes of individuals at target ages of 3, 6, 12, 24, 36 and 48 months (figure 1*b*; see electronic supplementary material, Information 1 for details of selection criteria). All *in vivo* models were prepared for the measurement of the anatomical CSAs of different masticatory muscle groups. The selected models at target ages were used to estimate muscle force vectors.

*In silico* finite-element models at 3, 6, 12, 24, 36 and 48 months of age were developed from the selected crania representing each target age. These skulls were initially reconstructed in Avizo, and then manually segmented into separated cranial bones and/or sutures. To capture the different degrees of suture patency during ontogeny [20,21], the 3 months model was segmented with major facial, calvarial and skull base joints (including synchondroses and fontanelles), the 6 months model was segmented with major calvarial sutures (including fontanelles), the 12 months model was segmented with anterior fontanelles only, and no sutures were segmented for the models representing 24, 36 and 48 months of age (figure 1*b* and electronic supplementary material, figure S1). Several simplifications were made during segmentation while ensuring adequate representation of key anatomical features (i.e. smooth and non-overlapped suture–bone interfaces) [39,40]. Subsequently, the segmented models were converted into three-dimensional solid meshes, consisting of 1.1 to 1.6 million tetrahedral elements according to model size, modified in HyperMesh software (V2022, Altair Engineering Inc., Troy, MI, USA), and then imported to a finite-element solver, ANSYS (V2022 R2, ANSYS Inc., Canonsburg, PA, USA).

### 2.3. Muscle anatomical cross-sectional areas and force magnitudes

**Figure 2.**
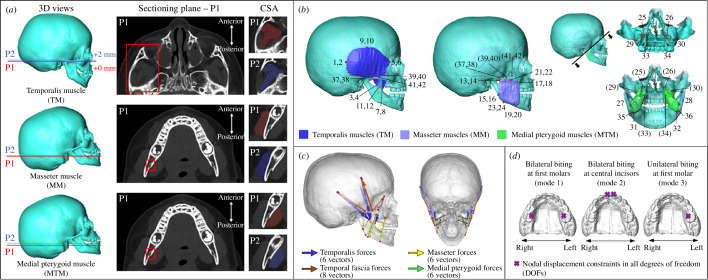
Identification of masticatory muscles and force vectors. (*a*) Maximum anatomical cross-sectional areas (CSA_max_) of the left temporalis, masseter and medial pterygoid muscles (TM, MM and MTM) were measured from CT images. All image stacks were aligned following different protocols for each muscle (first column; see electronic supplementary material, figure S2 for details of alignment protocols), the anatomical features for locating the first sectioning plane (P1) are highlighted in the second column (red boxes), P2 is parallel to and located 2 mm above P1, and CSA_max_ is the average of CSAs measured from two planes (third column). (*b*) Forty-two bilateral landmarks were used to mark up the origins and insertions of the temporalis muscle (nos. 1–12), masseter muscle (nos. 13–24), medial pterygoid muscle (nos. 25–36) and temporal fascia (nos. 37–42). (*c*) Force vectors of the temporalis (six vectors, in blue), masseter (six vectors, in yellow) and medial pterygoid muscles (eight vectors, in brown) as well as the temporal fascia (six vectors, in green). See definitions of each landmark and orientations of force vectors in electronic supplementary material, table S3. (*d*) Three simulated biting modes including bilateral biting at the first molars (mode 1), bilateral biting at the central incisors (mode 2) and unilateral biting at the left first molar (mode 3).

The procedures for measuring the maximum anatomical cross-sectional areas (CSA_max_) from CT scans to estimate peak muscle forces were based on Weijs & Hillen [31] and previous studies [3,8,9]. *In vivo* models (*n* = 51) were aligned following protocols to ensure that the orientations of the sectioning planes were perpendicular to the average fibre directions of temporalis, masseter and medial pterygoid muscles (figure 2*a*; see electronic supplementary material, figure S2 for alignment protocols). The reference sectioning plane (P1) for the temporalis was standardized using the CT slice with the completely visible zygomatic arch, in superior view (first row, figure 2*a*). For the masseter and medial pterygoid muscles, P1 was standardized at the level of the inferior part of the lingula on the mandible (second and third rows, figure 2*a*). The CSA_max_ of temporalis, masseter and medial pterygoid muscles were estimated as the average of the CSAs in P1 and in the second sectioning plane (P2, + 2 mm) immediately above it. Note that, all CSAs were measured using the left side muscles for consistency, as there was no obvious difference (< 5%) between both sides. The validity of using data from one side was assessed by comparing the muscle CSAs from both sides based on several randomly selected individuals throughout the studied age range (max. difference approx. 3%; electronic supplementary material, table S2). For each individual, temporalis, masseter and medial pterygoid peak muscle forces at each side were estimated by multiplying their CSA_max_ by a specific muscle stress factor (37 N cm^−2^) [8]. Temporal fascial force (TFF) was determined by carrying out a series of sensitivity tests through finite-element simulations (see below). Note that only the forces arising from the inferior attachment of the temporalis fascia were included in the models. The forces applied by the fascia to its upper, temporal attachments were omitted. This was mainly because we were interested in the level of strain in the facial region during the growth as calvarial growth is dominated by brain expansion (e.g. [14,15]). This simplification means that when TFFs were included in the model, biting forces were inevitably reduced because they act in an approximately opposite direction to bite forces, being unopposed by the forces that the temporal fascia would apply *in vivo* to the temporal lines.

### 2.4. Material properties, masticatory loading conditions and biting mode constraints

Isotropic (linear and elastic) material properties were assigned to all components. Based on prior literature [15,40,41], cranial bones and joints (including synchondroses and fontanelles) were assumed to have a baseline linear elastic modulus of 421 and 30 MPa, and corresponding Poisson’s ratio of 0.22 and 0.3 at 3 months of age, respectively. To replicate the tissue differentiation and changes in the bone and cranial joint properties during ontogeny, the elastic moduli of bone and cranial joints were increased by 125 MPa month^−1^ up to 48 months and 100 MPa month^−1^ up to 12 months [41,42].

In the simulation, we modelled the masticatory system loadings by applying forces at nodes where each muscle originates, oriented according to estimated muscle vectors based on their attachments, representing the maximal activation of each muscle (figure 2*b* and *c*). To identify masticatory muscle attachments, the temporalis, masseter and medial pterygoid muscles on both sides were fully segmented and reconstructed using the selected models at 3, 6, 12, 24, 36 and 48 months of age (i.e. segmented muscles at 48 months, figure 2*b*). However, because of limitations imposed by the resolution of the CT images (voxel sizes: 0.3–0.6 mm per direction; electronic supplementary material, table S1), we were not able to clearly identify the borders of the temporal fascia for segmentation. As the temporal fascial layer closely covers the temporalis muscles and is inserted along the superior border of the zygomatic arch [43], the origins and insertions of the temporal fascia were estimated as lying along the superior borders of the zygomatic arch and overlying the segmented temporalis muscle. Total of 42 landmarks were placed at the borders and in the middle of the origins and insertions of the temporalis (nos. 1–12), masseter (nos. 13–24), medial pterygoid (nos. 25–36) and temporal fascia (nos. 27–42) on both sides (figure 2*b* and see electronic supplementary material, table S3*a* for landmark definitions). There were six vectors (three vectors per side) defined for each temporalis, masseter and medial pterygoid muscle and eight vectors defined for the temporal fascia (figure 2*c* and electronic supplementary material, table S3*b*). The magnitude of the force applied to each muscle vector was calculated by dividing the estimated muscle force by the number of vectors on each side. The TFFs for each model were determined by a series of sensitivity tests, as detailed below.

Three biting modes were modelled, as shown in figure 2*d*, including bilateral biting at the first molars (mode 1), bilateral biting at the central incisors (mode 2) and unilateral biting at the first molar (mode 3). Nodal constraints against displacement in all directions (no rotational constraints) [22] were applied along the bite points of the first molars on both sides (mode 1), the central incisors on both sides (mode 2) or on the left-sided first molar (mode 3). Before dental eruption (< 12 months), the bite point was considered as being a point on the margin of the alveolus estimated to be the site of eruption of the first molar or central incisor. Once erupted, bite points were estimated as the cusps of the crown of the analysed teeth (12–48 months). The same translational constraints in all three axes were applied bilaterally to the paired nodes at the locations of the temporomandibular joint (TMJ; see electronic supplementary material, figure S3*a* for details of nodal locations). The impact of the choice of nodal constraints applied to the TMJs was assessed through a sensitivity test, indicating no significant differences (< 5%) in resulting bite forces and TMJ reaction forces between the applied approach and other conditions with reduced constraints [10,22] (electronic supplementary material, figure S3*b–h*). Furthermore, symmetric TMJ constraints may reduce the effects of normal asymmetry on the resulting reaction forces at TMJs [44] in the six independent skull models representing different ages.

### 2.5. Estimation of temporal fascial forces

Although several recent works have characterized the material properties of the temporal fascia in adult cadavers [45–47], data on TFFs in normal infants are not available from prior studies. Therefore, a series of standardized sensitivity analyses were performed to estimate the minimum force that needs to be applied by the temporal fascia to resist inferior deflection of the zygomatic arch during biting simulations at 3, 6, 12, 24, 36 and 48 months, using FEA (see electronic supplementary material, figure S4 for the details of whole sensitivity analyses). In brief, the TFFs applied to six models representing each age were scaled based on their effective zygomatic arch length and an estimation of force per millim, as detailed by Curtis *et al.* [10].

### 2.6. Model analysis

The bite forces under the three biting modes, with or without modelled temporal fascia, were estimated at 3, 6, 12, 24, 36 and 48 months of age as the reaction forces at the constraints applied to the bite points (cusps of the crown of the analysed teeth or margins of the alveolar bone). The maximum bite forces (from bilateral biting at first molars—mode 1) were then compared with experimental *in vivo* bite forces measured in the equivalent bilateral bites in healthy individuals aged from 3 to 7 years. The *in vivo* data were collected from the literature [4,23–27] as summarized in electronic supplementary material, table S4. Changes in strain and stress (von Mises) distributions across the entire craniofacial region (bones and joints) under masticatory system loadings throughout the sampled age range were compared between models with and without applied temporal fascia loads. Further quantification focused on the nodal strain and stress changes at nine specific locations, including the (i) lateral inferior forehead, (ii) *nasion*, (iii) *alare*, (iv) upper part of the frontal process of maxilla, (v) lateral process of maxilla, (vi) inferior orbital roof, (vii) upper frontal process of zygoma, (viii) central zygoma, and (ix) central palate.

## 3. Results

### 3.1. Masticatory muscle cross-sectional areas and forces

**Figure 3.**
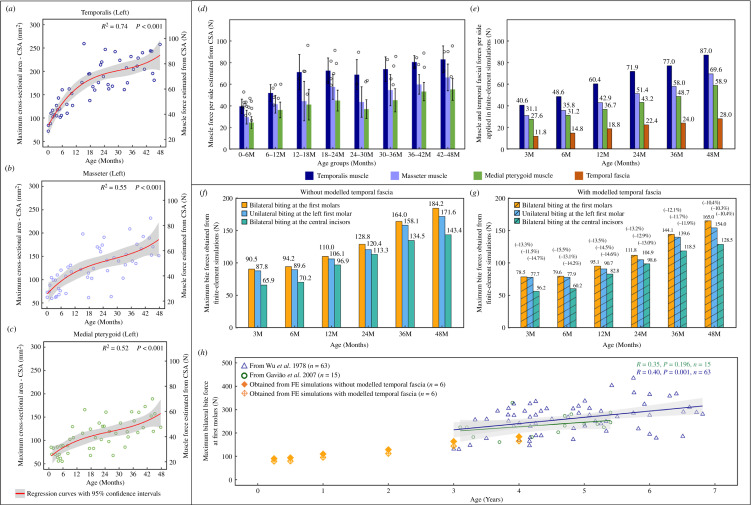
Estimated muscle forces and predicted biting forces. (*a*–*c*) Maximum muscle forces per side of the temporalis (*a*), masseter (*b*) and medial pterygoid (*c*) estimated by multiplying the CSA_max_ and a constant muscle stress factor (37 N cm^−2^). The mean estimated maximum muscle forces per side of each age group are presented in (*d*). (*e*) Forces applied by the temporalis (in blue), masseter (in purple), medial pterygoid muscles (in green) and the temporal fascia (in brown) to the six *in silico* models. The muscle forces of the temporalis, masseter, and medial pterygoid at each age were calculated from the regression functions (*a*, in red) based on the 51 sampled crania, while the forces applied by the temporal fascia at the same ages were determined through sensitivity tests (electronic supplementary material, figures S4–S10 and electronic supplementary material, table S5). The predicted total bite forces under three biting modes at specific ages without modelled temporal fascia (*f*) and with modelled temporal fascia (*g*), and the percentages shown in (*g*) indicate the relative differences in bite forces compared with the corresponding value in (*f*). (*h*) Comparison between the predicted and *in vivo* maximum bite forces (bilateral biting at the first molars; see electronic supplementary material, table S4 for more details of *in vivo* bite force data). Note that all regression curves are reported with 95% confidence intervals (in grey).

The changes in the measured maximum cross-sectional areas (CSA_max_) of the temporalis, masseter and medial pterygoid muscles as well as the peak forces were estimated from these between 0 and 48 months in figure 3*a*–c. Based on regression, the mean temporalis muscle force increases nonlinearly from 30.9 N at 3 months to 87.0 N at 48 months (min. to max.: 26.6 to 96.0 N) on each side. The temporalis muscle peak force undergoes a rapid increase in the first 18 months then increases more slowly until 36 months before accelerating again (figure 3*d*). The mean peak forces of the masseter and medial pterygoid muscles increase from 25.6 to 69.6 N (min. to max.: 22.2 to 86.0 N) and 23.1 to 58.9 N (min. to max.: 17.5 to 70.0 N), respectively, following a similar trend to that of the temporalis throughout the sampled age range (figure 3*d*). Considering the individual variances and sample distributions (figure 3*d*), the peak forces to be applied to the six *in silico* models by the temporalis, masseter and medial pterygoid muscles were calculated at each age from the nonlinear regression functions (figure 3*e*). TFFs magnitudes estimated as described above (and see electronic supplementary material, table S5, electronic supplementary material, figures S4–S10) were applied to the same models. The minimum balanced fascia forces at the ages of 3, 6, 12, 24, 36 and 48 months are around 30% of temporal muscle force and around 42% of masseter muscle force at the same age, as presented in figure 3*e*.

### 3.2. Predicted *in silico* bite force, temporomandibular joint reaction force and comparisons with published data

When applying the loadings of the temporalis, masseter and medial pterygoid muscles with no modelled temporal fascia, the *in silico* bite forces under the three simulated biting modes change in similar ways over time, maintaining very similar force magnitudes until 6 months and then undergoing a rapid and linear increase from 12 to 48 months (figure 3*f*). Maximum bite force (bilateral biting at the first molars—mode 1) increases from 94.2 N at 6 months to 184.2 N at 48 months. The maximum bite force under unilateral molar biting (mode 3) is similar to the values obtained from the bilateral biting until 6 months, but by 48 months, it is approximately 7% less. The maximum bilateral biting force at the central incisors (mode 2) is lower, approximately 73–88% of the maximum biting force under bilateral biting at first molars at the same ages (figure 3*f*). When TFFs are applied to the zygomatic arch (but no reaction force is applied to the temporal lines, see §2), as expected the estimated maximum biting forces under each of the three biting modes are reduced by 10–15%, but follow the same trend with age (figure 3*g*).

Mean *in vivo* bite forces at the first molars have been reported to be 196.0 ± 96.1 N (*n* = 15) [24] and 213.2 ± 44.0 N (*n* = 30) [25] from 3 to 5 years, 235.1 ± 44.5 N (*n* = 15) [23] from 3 to 5.5 years and 262.4 ± 66.4 N (*n* = 63) [27] from 3 to 7 years. Our *in silico* maximum bite forces estimated at younger ages are lower, 90.5 N (no TFFs) and 78.5 N (with TFFs) at 3 months increasing to 184.2 N (no TFFs) and 165.0 N (with TFFs) at 48 months. The estimated bite forces from 36 to 48 months temporally overlap the published experimental data and lie within the lower range of bite force values. From figure 3*h*, it is evident that our *in silico* estimates of bite force increase with age at a similar rate to the two *in vivo* datasets (reported with individual bite forces) [23,27], but have generally lower values (approximate 21–32%).

Furthermore, considering the correlations with applied muscle forces and resulting bite forces [44], the maximum reaction forces at the TMJs, under the three simulated biting modes and with modelled temporal fascia, were measured and are reported in electronic supplementary material, table S6. When biting bilaterally, the total TMJ reaction forces (measured at two constrained nodes per side) are similar between left and right sides, and the mean reaction force per side increases from 36.8 to 86.8 N (at the first molars—mode 1) and from 47.1 to 108.6 N (at the central incisors—mode 2), from 3 to 48 months. When biting unilaterally (at the left side first molar—mode 3), the reaction force at the balancing (right) side increases from 48.6 to 120.1 N over the first 48 months of age, which is more loaded than that of working (left) side (from 34.7 to 65.9 N).

### 3.3. Changes in cranial strain and stress distributions

**Figure 4.**
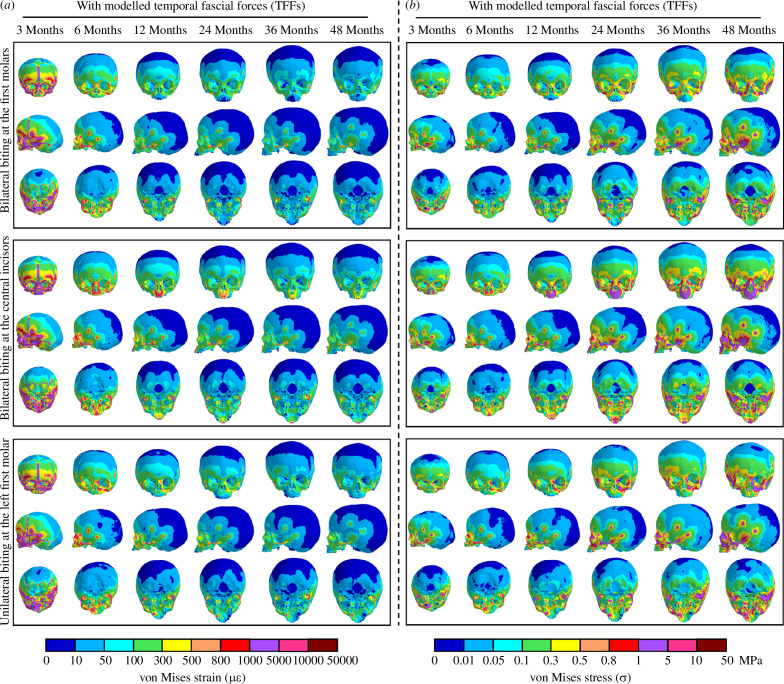
Contour plots of von Mises strains (*a*) and stresses (*b*) over the skull models at 3, 6, 12, 24, 36 and 48 months of age under three simulated biting modes with temporal fascia loads, reported in anterior, sagittal and dorsal views. Note that non-uniform scale bars have been applied to accommodate the wide range of strain and stress magnitudes across regions and ages. The corresponding results modelled without temporal fascia loads are attached in electronic supplementary material, figure S11.

**Figure 5.**
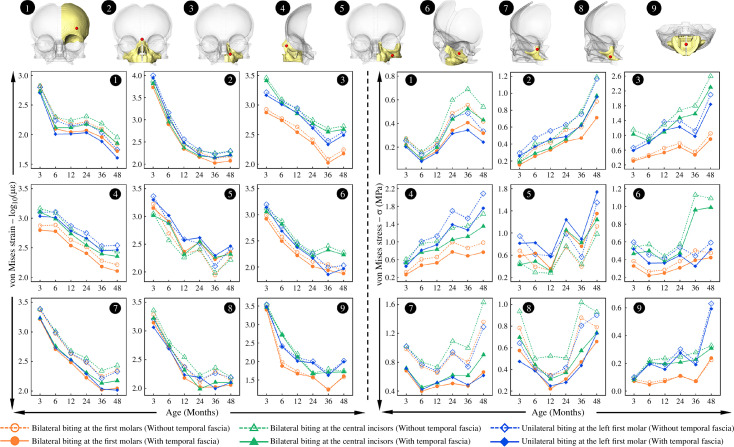
Comparisons of nodal von Mises strain and stress extracted at nine specific locations on the facial region at 3, 6, 12, 24, 36 and 48 months under all simulated loading conditions. The locations (in red) are indicated on three-dimensional models in the top row: (1) lateral inferior forehead; (2) *nasion*; (3) *alare*; (4) upper part of the frontal process of maxilla; (5) lateral process of maxilla; (6) inferior orbital roof; (7) upper frontal process of zygoma; (8) central zygoma; (9) central palate. Note that all strain values are reported after natural logarithm transformation (log_10_) to accommodate the large changes in magnitude.

Strain and stress patterns across the crania with all masticatory loading conditions under three biting modes at 3, 6, 12, 24, 36 and 48 months are presented in figure 4 (with TFFs) and electronic supplementary material, figure S11 (without TFFs), while the magnitudes of von Mises strain and stress at specific locations on the mid-face are shown in figure 5.

When biting bilaterally at the first molars (leading to the maximum predicted bite force among considered load cases), the overall level of mechanical strain (von Mises) across the crania (including both bones and cranial joints) decreases with age, and the largest magnitude of von Mises strain in the facial region changes from 10 000 µε to 100 µε from 3 to 48 months of age. Consistently, predicted strains over the calvaria and skull base, both without and with modelled temporal fascia loads, are lower than in the face (first row, figure 4*a*). From the anterior view, the entire face from the forehead to upper palate (including the metopic, coronal and major facial sutures) experiences high levels of von Mises strain (> 1000 µε) at 3 months; subsequently, the region that is relatively highly strained is reduced to the mid-face (from upper orbital rim to lower maxilla) with strains reduced to around 300 µε on average at 24 months. By 48 months (first row, figure 4*a*), the largest predicted strains are limited to the zygomatic and maxillary regions. In sagittal view, von Mises strains across the calvaria (including the sagittal and lambdoid sutures) decrease rapidly from around 100 µε to 10 µε in the first 12 months and then remain relatively unchanged (< 10 µε) until 48 months (first row, figure 4*a*). From the inferior view, the strains over the upper palate, zygomatic arches and sphenoid change similarly (decreasing in the first 12 months then remaining approximately unchanged). A similar trend is seen in the skull base, reducing from relatively high strains in the first 6 months (100–800 µε) to a more consistent lower magnitude of strain (10–50 µε) from 12 to 48 months (first row, figure 4*a*). Between the models with and without modelled temporal fascia loads, there are no obvious differences in strain distributions over the calvaria and skull base, but the addition of TFF balances the zygomatic arch, leading to uniform strains across its length (figure 4*a* and electronic supplementary material, figure S11*a*) and reduced overall level of strain across most of the mid-facial region by approximately 50 µε (left panel, figure 5). Moreover, strain magnitudes over the fronto-zygomatic region (i.e. points 1, 7 and 8), as well as the upper nasal region (point 2), are reduced to 110 µε, while only the lateral maxillary process (point 5) experiences the same or slightly higher magnitudes of von Mises strain when incorporating the TFFs (left panel, figure 5). The strain distribution over the crania also differs among the three simulated biting modes. Bilateral biting at the first molars leads to large strains over the zygo-maxillary regions during infancy (first row, figure 4*a*), bilateral biting at the central incisors generally increases the strains from the upper nasal to mid palatal regions (including the lateral nasal wall and the palate; second row, figure 4*a*), while unilateral biting at the first molar leads to an asymmetric distribution of strains, especially in the face, with the nasal, maxillary and zygomatic regions on the working side under higher tension than other areas (third row, figure 4*a*). In the models representing the youngest individuals in which sutures are modelled, compared to biting at the central incisors, biting at the first molars unilaterally and bilaterally leads to greater strains in the lambdoid sutures but lower strains in skull base joints (figure 4*a*).

Stress distributions over the crania during infancy change with age in the opposite way to strains, Thus, in general, mechanical stresses (von Mises) increase from 3 to 48 months under all simulated loading conditions (figure 4*b* and electronic supplementary material, figure S11b). At 3 months, under bilateral first molar biting (first row, figure 4*b*), the greatest stresses (> 0.5 MPa) are found over the upper nasal region, inferior zygomatic border and posterior zygomatic process. Subsequently, regions of high stress extend over the mid-lateral zygo-maxillary regions by 24 months, and then over much of the facial skeleton (including all facial sutures) until they cover the entire mid-face by 48 months. The posterior cranial vault (including the sagittal and lambdoid sutures) remains under more or less constant low levels of stress (< 0.05 MPa), while the entire skull base (including synchondroses) and forehead (including the metopic suture) experience an intermediate magnitude of stress (between 0.1 and 0.3 MPa from 3 to 48 months). The effect of including temporal fascial loads is to reduce the overall stress magnitudes over the mid-face (except at the lateral process of maxilla; point 5, right panel, figure 5) by around 0.2 MPa on average, while the stresses recorded at several specific locations (points 1, 2, 7 and 8, right panel, figure 5) decrease by a maximum of 0.7 MPa during infancy. Moreover, stress distributions differ in the same way among the three biting modes, as was noted for strains. All three biting modes result in increasing stress magnitudes over inferior orbital region, orbital rims, orbital roof and the lateral aspect of the nasal region (figure 4*b*).

## 4. Discussion

### 4.1. Masticatory muscle force changes

We found that the overall changes in the estimated peak muscle forces of the temporalis, masseter and medial pterygoid muscles are related to the accelerations and decelerations of cranial size changes over the first 48 months of the whole available samples (*n* = 217, figure 1*b*). In turn, skeletal size changes are to a large degree driven by the growth of various soft tissues and organs (i.e. brain, eyes, nose and tongue), interacting with muscle actions, as posited in functional matrix theory [48,49] and partially tested by our previous study [14]. Moreover, how estimated force changes during growth vary among masticatory muscles. Thus, brain expansion dominates early calvarial growth and brain volume increases rapidly in the first 12 months, then slows down until 36 months before undergoing another accelerated phase [14]. Meanwhile, it is interesting that the temporalis muscle force, which is attached to the calvarium, changes over time in a similar way to the intracranial volume (see fig. 3A of reference [14]) over the first 48 months (figure 3*a*). On the other hand, masseter and medial pterygoid muscle forces follow a more linear trajectory of increase (figure 3*b* and *c*) compared with the temporalis, and this might be linked to the fact that facial growth follows a more linear trajectory, partly driven by the linear expansion of the cartilage and soft tissues of the nasal and palatal region over the same period (see fig. 3E,G of reference [14]). Since peak muscle forces are directly calculated from muscle CSA_max_, variations in estimated forces directly reflect changes in muscle CSAs.

### 4.2. Bite force and temporomandibular joint reaction force changes

The predicted *in silico* maximum bite forces (figure 3*f,* *g*) were slightly lower than bite forces measured in the previous *in vivo* studies [23–27] examining biting between 36 and 48 months (figure 3*h*). This could be due to a number of reasons, e.g. differences in the ethnicity of the populations in which bite forces were measured in prior *in vivo* studies compared to this study, or it could be due to the simplification of using muscle CSA to estimate physiological CSA and, therefore, muscle peak force, given the complex internal architecture of at least some masticatory muscles (e.g. pennation in masseter). Nonetheless, our modelling approach enables us to estimate the bite force capabilities of children at younger ages and to predict stresses and strains over the cranium at earlier ages.

Predicted bite forces change little during the first 6 months and then increase more or less linearly to 48 months, in models both with and without modelled temporal fascia. This finding reflects the changes in feeding that infants undergo in the first year of life. Thus, from 0 to 6 months, infants barely bite but rather rely on suckling [50,51], which likely places only small loads on the masticatory system and is reflected by the low peak forces achieved by the muscles of mastication and by the low bite forces achieved by our models representing this period. As the central incisor and first molar erupt at 10 and 16 months in average [28,29], infants start to bite and chew more solid foods and gradually progress to a diet more typical of adults by 24 months [30]. During this post-weaning phase, the masticatory muscle groups are activated and loaded at higher frequency and more commonly in biting. The linear increases in muscle CSAs, peak muscle forces and bite forces after 6 months reflect and enable these changes.

The bite forces arising from different biting modes depend on the locations of bite points in relation to the TMJs, muscle forces and their lever arms. On the one hand, over the first 48 months, the bite force is proportional to the symmetrically applied muscle forces (figure 3), and the TMJ reaction force also varies in the same way in response to increasing masticatory loads (electronic supplementary material, figure S3*c–h*). Such relations among bite, muscle and TMJ reaction forces have also been observed in normal adults [44], hence, our findings suggest consistency regarding these covariations throughout ontogeny. On the other hand, the infant cranium grows in both shape and size, resulting in changes in lever mechanics that contribute to our predicted changes in bite forces. For instance, unilateral or bilateral first molar biting produces a similar total bite force below 6 months but these come to differ with age as the face changes in proportions from a relatively wide face with small palate at birth to a relatively taller face with an anteroposteriorly longer palate. It grows more in height than breadth, and these changes are associated with, and to some extent driven by, expansion of the nasal region and oral soft tissues [4,5,14].

### 4.3. Mechanical significance of the temporal fascia

A key component of craniofacial growth comprises the adaptation of skeletal tissues to their mechanical environment, in particular to the strains experienced by cranial skeletal structures, which in turn, appear to influence surface remodelling [48,49,52–54]. Therefore, to fully model the impacts of mechanical loading on the growth of the skull, it is necessary to simulate both the direct effects of expansion of soft tissues on the relative displacements of the skeletal elements [14] and the indirect effects of the growth changes in the muscles, dentition and skeletal proportions on the development of skeletal strain and stress. Predictions of the changing mechanical strains and stresses experienced by each region of the cranium during growth might in turn be applied to predictions of craniofacial remodelling. Accurate prediction of the distributions and magnitudes of strains and stresses depends on, among other factors, accurate modelling of cranial size and shape, loading and tissue mechanical properties. While most FEA models of the human cranium [12,13,22,32–34,37] include skeletal and muscular components, they tend to overlook potentially important fascial elements. The associated temporal fascia, which overlies the temporalis muscle [11], is potentially important in this regard. It has a potential role in counteracting the action of the masseter on the zygomatic arch and, therefore, in modifying the functioning of, and strains experienced by, the zygomatic arch [10,55]. As such, we investigated the effects on the strains experienced by the zygomatic region during ontogeny. However, these forces are unknown and so, in this study, we describe an approach to estimate the TFFs required to balance (limit downward deflection) the zygomatic arch [10] through a series of sensitivity analyses (detailed in the electronic supplementary material, figures S4–S10). Such an approach is potentially useful in a wide range of studies [12,13,22,32–34,37,56], which have until now not been able to take TFFs into consideration.

Our results indicate that, in infants, the minimum TFF to be applied to the arch is perhaps around 42% of the magnitude of the applied masseter force. Also, predicted bite forces are 10–15% lower in our models with these TFFs applied to the upper aspect of the zygomatic arch (figure 3*f* and *g*). However, this reduction in force is likely to be an artefact of our modelling approach, in that we did not apply an equal and opposite total force to the temporal lines, which results in unloading of the bite point by the applied TFF. This can be expected to affect the local magnitudes but not the predicted pattern of strains.

Considering the effect of the TFFs applied to the zygomatic on the predicted levels of strain and stress: (i) no obvious difference was found in the calvarium and the skull base and (ii) the level of strain was considerably impacted in the zygomatic arch (figure 4 and electronic supplementary material, figure S11) but to a lesser degree across the mid-facial region (*ca.* 50 με, figure 5) and fronto-zygomatic region (*ca.* 110 με, figure 5). These findings suggest that the impact of modelling the fascia is limited to its attachment sites, and if we would have included reaction of the temporal fascia on the parietal bone, it would likely have had a localized effect across its attachment areas (as it did across the zygomatic arch). Therefore, while soft tissues such as the temporal fascia impact the predicted magnitudes and spatial distributions of stresses and strains in cranial finite element models, they may have a minimal effect on the overall results, depending on what is being investigated (e.g. [10,44,57]).

### 4.4. Functional adaptation of crania

As noted earlier, the mechanical stimuli arising from chewing and suckling in infants can impact metopic suture closure [22] and the timing and rates of suture closure significantly affect craniofacial growth and development [2,20,21,58]. Our results, provide insights into the impact of masticatory system loading on the macroscopic changes in craniofacial sutures over the first 48 months of life (figures 4 and 5). Besides the metopic suture, mechanical loads arising from the masticatory system also contribute to the closure of several large calvarial sutures [2] (including the coronal, squamosal and lower lambdoid sutures) as well as the skull base joints [20]. These neurocranial sutures tend to experience relatively high degrees of tension at 3 months. However, from 6 months onwards, our results suggest that strain induced across the calvarial sutures owing to the masticatory system loading are considerably reduced. Nonetheless, facial sutures remain patent to varying degrees [21]. It has been argued that the mechanical stresses and strains in facial sutures arising from masticatory system loading contribute to the growth of the bones adjacent to the sutures and their eventual ossification [17,19]. Our simulations indicate that the three considered bite modes led to different levels of mechanical strain across the sutures. Thus, compared with biting on the first molars, bilateral biting on the central incisors results in less tension across the lambdoid sutures and greater tension and stress on sutures located around the basioccipital (i.e. *posterior* intraoccipital suture).

Masticatory system loading results in higher levels of mechanical strain in both the facial bones and the skull base than in the calvarial bones (figure 4). This is evident across all considered ages, but more after 6 months of age. These findings are consistent with the hypothesis [59] that masticatory forces are a key driver of facial growth while the brain is the main driver of calvarial growth. This study quantified the levels of mechanical stress and strain in the face resulting only from masticatory system loading. Additionally, the eruption of the teeth and expansion of soft tissues [60,61] also contribute to the growth of the face, but there are very limited quantitative data relating to their contribution to the mechanics of craniofacial growth.

### 4.5. Limitations

This work has limitations; perhaps, the key ones are (i) the muscle forces of the temporalis, masseter and medial pterygoid were estimated based on CSAs measured from CT scans. However, for the studied age range, there is a lack of available *in vivo* electromyogram (EMG) data on masticatory muscle forces [62] for comparison and validation. (ii) Although our *in silico* bite forces are consistent with published *in vivo* experimental data between 36 and 48 months of age, data on bite forces before 36 months of age are limited, because of the ethical and technical difficulties of obtaining such data. (iii) Due to the limitations imposed by the resolution of clinical CT, each muscle group was segmented and measured as a whole, omitting details of internal anatomy (i.e. the deep and superficial masseters are treated as a single muscle). Additionally, peak muscle forces were estimated from CSA, ignoring details of the internal muscle architecture such as pennation. These likely contributed to underestimation of peak muscle forces. (iv) Linear and isotropic material properties were allocated to our *in silico* models, which is a commonly applied simplification of reality (e.g. [10,12,15,22,34,39,41,54,63]). Additionally, it is likely important to model the nonlinearities of the material behaviour of soft tissues (i.e. sutures) [7,64]. This was not possible in this study because of resource limitations, but the intention is to approach this issue in future studies. (v) The key craniofacial organs, such as the brain and eyes [14], were not modelled here and the biomechanical interactions between the masticatory system loads and these organs will be investigated in future work. (vi) Our modelling focuses only on biting, while the craniofacial system is loaded in diverse ways during food processing. Future studies should consider a wider range of masticatory system loadings (e.g. in chewing) and the impact of other physical parameters (e.g. occlusal area and variations in dental loading vectors).

## 5. Conclusion

This study predicts the development of bite forces in infants over the first 48 months of life and the magnitudes and distributions of mechanical strain and stress experienced by crania from the masticatory system biting loads during postanal growth. Our findings provide insights into the potential role of TFFs in balancing the zygomatic arch and their effect on strain distributions and the potential effects of masticatory system loading on the closure of the metopic, coronal and other cranial sutures during the first 12 months. Together, these analyses contribute to the future development of simulations of craniofacial growth driven solely by mechanical forces arising from the growth of soft tissues and the actions of muscles. Such simulations can be expected to be informative in assessing the role of mechanics in driving and modulating craniofacial growth and adaptation and they are likely to be applicable to the prediction of normal and pathological (e.g. craniosynostosis) growth.

## Data Availability

Previously published data were used for this work [14]. Supplementary material is available online [65].
